# A novel miR-34a target, protein kinase D1, stimulates cancer stemness and drug resistance through GSK3/β-catenin signaling in breast cancer

**DOI:** 10.18632/oncotarget.7443

**Published:** 2016-02-17

**Authors:** Do Yeon Kim, Eun Young Park, EunSun Chang, Hyeok-Gu Kang, Yoonjin Koo, Eun Ji Lee, Je Yeong Ko, Hyun Kyung Kong, Kyung-Hee Chun, Jong Hoon Park

**Affiliations:** ^1^ Department of Biological Science, Sookmyung Women's University, Seoul, Republic of Korea; ^2^ Department of Biochemistry and Molecular Biology, Yonsei University College of Medicine, Seoul, Republic of Korea; ^3^ Brain Korea 21 PLUS Project for Medical Science, Yonsei University College of Medicine, Seoul, Republic of Korea

**Keywords:** miR-34a, PRKD1, β-catenin signaling, cancer stemness, drug resistance

## Abstract

One of the properties of human breast cancer cells is cancer stemness, which is characterized by self-renewal capability and drug resistance. Protein kinase D1 (*PRKD1*) functions as a key regulator of many cellular processes and is downregulated in invasive breast cancer cells. In this study, we found that *PRKD1* was upregulated in MCF-7-ADR human breast cancer cells characterized by drug resistance. Additionally, we discovered that *PRKD1* expression was negatively regulated by miR-34a binding to the *PRKD1* 3′-UTR. *PRKD1* expression increased following performance of a tumorsphere formation assay in MCF-7-ADR cells. We also found that reduction of *PRKD1* by ectopic miR-34a expression or *PRKD1* siRNA treatment resulted in suppressed self-renewal ability in breast cancer stem cells. Furthermore, we confirmed that the *PRKD1* inhibitor CRT0066101 reduced phosphorylated PKD/PKCμ, leading to suppression of breast cancer stemness through GSK3/β-catenin signaling. *PRKD1* inhibition also influenced apoptosis initiation in MCF-7-ADR cells. Tumors from nude mice treated with miR-34a or CRT0066101 showed suppressed tumor growth, proliferation, and induced apoptosis. These results provide evidence that regulation of *PRKD1*, a novel miR-34a target, contributes to overcoming cancer stemness and drug resistance in human breast cancer.

## INTRODUCTION

Breast cancer is the most common type of cancer and the leading cause of cancer-related death in women worldwide [1]. Despite efforts to improve patient survival rates, problems associated with breast cancer therapy, including cancer metastasis and drug resistance, remain [2, 3]. Tumors are organized with cancer stem cells (CSCs) and non-tumorigenic cells forming a tumor mass [4]. CSCs are considered the cause of tumors, cancer metastasis, drug resistance, and cancer relapse [5]. Specifically, CSCs in breast cancer (BCSC) display stem-cell properties and are characterized by expression of the cell-surface marker CD44+/CD24- [6]. Different miRNAs are involved in the formation and regulation of human BCSCs [7], with previous studies reporting that ectopic expression of miR-34c suppressed epithelial–mesenchymal transition and reduced self-renewal capacity in BCSCs [8].

The serine/threonine-protein kinase D1 (*PRKD1*) functions as diacylglycerol and protein kinase C (PKC) effectors that mediate the actions of stimuli [9]. Processes associated with protein kinase D (PKD)/PKCμ were activated by two phosphorylation loops through PKC-dependent phosphorylation (Ser744/Ser748) and PKC-independent autophosphorylation (Ser910) [10-13]. Therefore, *PRKD1* is considered as a key regulator of many cellular processes, including initiation of the NF-kB signaling pathway, enhancement of cell cycle progression and DNA synthesis, and regulation of other pathological conditions [14-16].

MicroRNA regulates apoptosis, tumorigenesis, and angiogenesis in breast cancer. A key regulator of tumor suppression, miR-34 is a direct transcriptional target of the tumor suppressor p53, given that the miR-34a promoter region contains a p53-binding site [17]. In breast cancer studies, miR-34a played a role in preventing cell survival by upregulating p53 post-irradiation after DNA had been damaged [18]. Additionally, miR-34a promoted cancer-cell apoptosis by targeting Bcl-2 and SIRT1 [19]. Therefore, miR-34a may be associated with targets that induce breast cancer.

In this study, we found that overexpressed *PRKD1* was inhibited by miR-34a in MCF-7-ADR cells. Furthermore, *PRKD1* activated the self-renewal capacity in BCSCs through glycogen synthase kinase 3 (GSK3)/β-catenin signaling, and contributed to the elimination of drug resistance. These results suggest important roles for *PRKD1*, a novel miR-34a target, in human breast cancer therapy.

## RESULTS

### miR-34a suppresses PRKD1 in MCF-7-ADR cells

**Figure 1:**
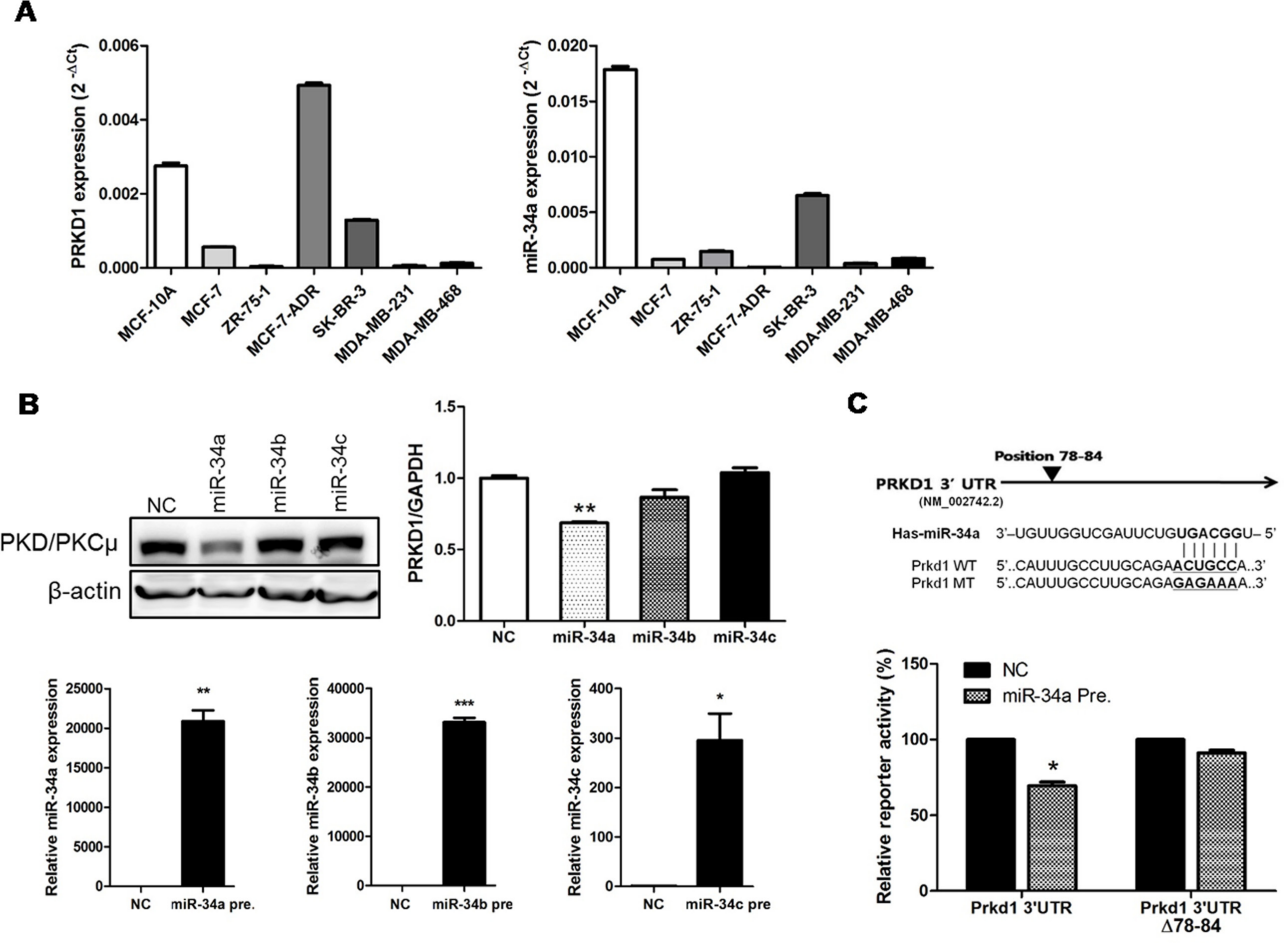
*PRKD1* is a novel miR-34a target **A.** *PRKD1* mRNA expression and miR-34a expression was quantified by qRT-PCR in various breast cancer cell lines. **B.** Proteins, mRNAs and totalRNAs were obtained after 48-h transfection of miRNA-34 variants. Western blots are representative of three independent experiments. β-actin was used as the loading control and qRT-PCR was performed to validate *PRKD1* mRNA and miR-34 variant expression. The levels of miR-34a, b and c expression were detected following ectopic expression of miR-34a, b and c, respectively. **C.** Predicted miR-34a binding site and reporter constructs from the wild-type/mutant *PRKD1* 3′-UTR. The activities of the 3′-UTR reporter constructs were normalized to the activity of co-transfected phRL-Luc vector. The graphs show mean ± S.D. (error bars) from three independent experiments. * *p* < 0.05; ** *p* < 0.001; *** *p* < 0.0001.

We evaluated *PRKD1* expression in breast cancer cell lines, including MCF-10A, MCF-7, ZR-75-1, MCF-7-ADR, SK-BR-3, MDA-MB-231, and MDA-MB-468. The results indicated increased *PRKD1* expression levels in MCF-7-ADR cells (Figure 1A). We determined possible miRNAs capable of regulating *PRKD1* by using microRNA prediction online databases [miRanda (http://www.microrna.org/microrna/home.do) and TargetScan (http://www.targetscan.org/)]. Given that miR-34 was a candidate regulator, we determined *PRKD1* mRNA expression and protein translation levels following ectopic expression of miR-34a, miR-34b, and miR-34c. Although miR-34a, miR-34b, and miR-34c have the same seed sequence, the results indicated that *PKD/PKCμ* was downregulated only by miR-34a (Figure 1B). To confirm the miR-34a binds to the *PRKD1* 3′-UTR, we mutated the predicted miR-34a binding site on the *PRKD1* 3′-UTR and inserted the mutated sequence into a pGL3-control vector (Figure 1C). As shown in Figure 1C, overexpression of miR-34a inhibited the luciferase activity of the *PRKD1* wild-type sequence, but not that of the mutants in MCF-7-ADR cells. We screened for the levels of miR-34a expression in breast cancer cell lines, and consistent with the results shown in Figure 1A, miR-34a was downregulated in MCF-7-ADR cells. These results indicate that miR-34a negatively regulates *PRKD1* (Figure 1A).

Expression levels of miR-34b and miR-34c were also detected, however, no significant downregulation of either variant in MCF-7-ADR cells was observed (Supplementary Figure 1A, 1B). These results suggest that *PRKD1* is downregulated by miR-34a in MCF-7-ADR cell lines.

### PRKD1 stimulates breast cancer stemness through GSK3/β-catenin signaling

**Figure 2:**
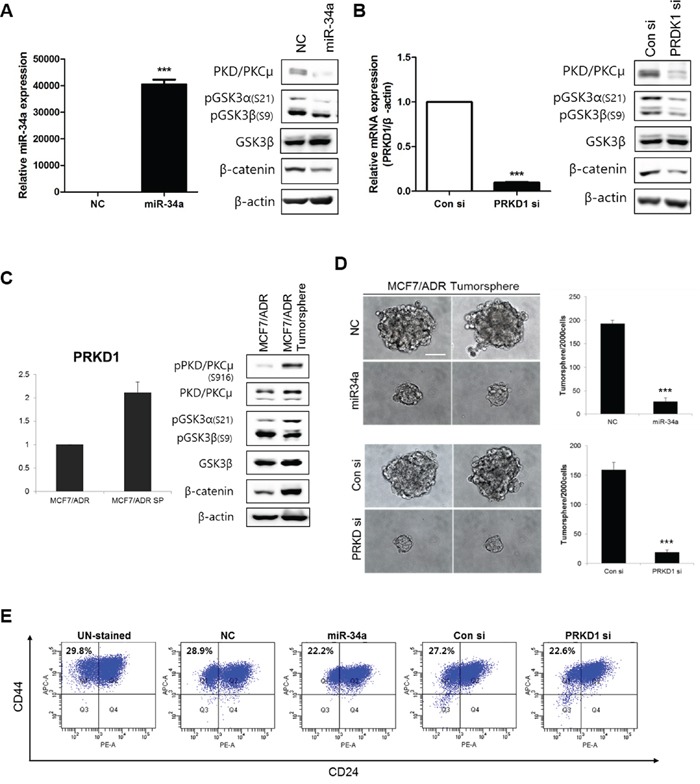
Effects of PKD/PKCμ downregulation on breast cancer stemness through GSK3/β-catenin signaling in MCF-7-ADR cells All results were obtained from more than five independent transfection experiments. **A-B.** miR-34a precursor and *PRKD1* siRNAs (15 nM) were transfected into MCF-7-ADR cells and levels of miR-34a and *PRKD1* expression confirmed by qRT-PCR. Western blot analysis of GSK3/β-catenin signaling. β-actin was used as the loading control. **C.** Basal phosphorylation and expression of PKD/PKCμ in tumorspheres from MCF-7-ADR cells as compared with two-dimensional cultured MCF-7 cells. **D.** Representative confocal images of tumorsphere formation were captured using an Olympus IX71 at a magnification of 400×. Scale bars represent 50 μm. **E.** Analysis of the cell-surface expression of mammary stem cell markers in MCF-7-ADR cell cultures. Histograms represent results of five independent experiments. Percentages indicate the number of cells in that quadrant. Bars represent each sample performed in triplicate and the error bars represent the ± S.D. * *p* < 0.05; ** *p* < 0.001; *** *p* < 0.0001.

To determine the effects of *PRKD1* inhibition on CSCs, MCF-7-ADR cells were transfected with miR-34a precursors and *PRKD1* siRNAs. Following transfection, miR-34a expression levels increased and PKD/PKCμ levels decreased relative to negative control (Figure 2A). *PRKD1* expression levels also decreased following *PRKD1* siRNA transfection as compared to levels observed in association with transfection of control siRNA. Interestingly, PKD/PKCμ levels also decreased following *PRKD1* siRNA transfection (Figure 2B). We checked efficiency of three different *PRKD1* siRNAs to exclude unspecific effects and we selected PRKD1 siRNA #1, which was shown high suppression of *PRKD1* relative with the others (Supplementary Figure 2).

*PRKD1* phosphorylation of β-catenin at Thr112/Thr120 could be critical for cell-cell adhesion in prostate cancer cells [20]. Furthermore, a complex of CDC42, PAR6, and PKCζ binds GSK3β and catalyzes the phosphorylation of Ser9 to inhibit GSK3β [21]. In order to associate *PRKD1* with GSK3/β-catenin signaling, we performed western blot analysis. The results showed that reduction of PKD/PKCμ suppressed β-catenin expression and GSK3α and GSKβ phosphorylation (Figure 2A). These results were confirmed in control and *PRKD1* siRNA-treated cells (Figure 2B). In addition, we confirmed that miR-34a expression levels were decreased by altering GSK3/β-catenin signaling following transfection with miR-34a inhibitors in MCF-7 cells, as a control (Supplementary Figure 3A, 3B).

*PRKD1* expression and GSK3/β-catenin signaling were upregulated in MCF-7-ADR cells exhibiting tumorsphere formation (Figure 2C). Cancer stemness markers such as *OCT4* and *SOX2* were highly expressed in MCF-7-ADR cells in sphere status (Supplementary Figure 4A, 4B). Furthermore, we also observed the expression of miR-34a was lower and the expression of *PRKD1* was higher in MCF-7-ADR mammospheres compared with MCF-7 mammospheres (Supplementary Figure 5A, 5B). To investigate the effects of *PRKD1* knockdown in breast cancer stemness, we performed a Tumorsphere formation assay. *PRKD1* knockdown by miR-34a precursors and *PRKD1* siRNA significantly decreased the number of tumorspheres (>70 μm) relative to controls (Figure 2D). Additionally, the BCSC population was determined by fluorescence-activated cell sorting analysis with staining for the BCSC markers CD44+/CD24-. The CD44+/CD24- population (Q1) was reduced by *PRKD1* knockdown (Figure 2E). Collectively, *PRKD1* was capable of regulating cancer stemness in MCF-7-ADR cells by altering GSK3/β-catenin signaling.

### Inhibition of PKD/PKCμ phosphorylation reduces BCSC self-renewal capacity

**Figure 3:**
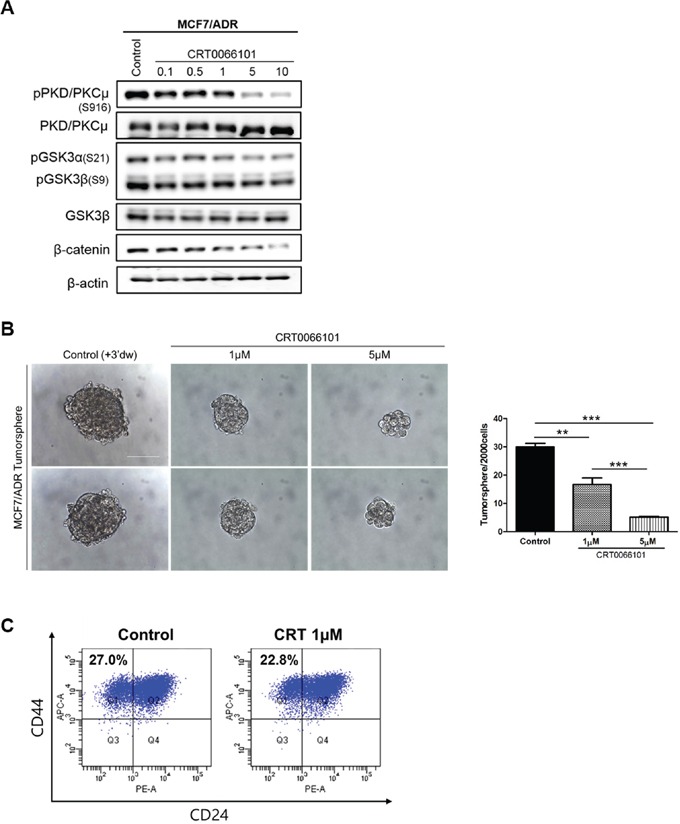
Effects of CRT0066101 on breast cancer stemness through GSK3/β-catenin signaling in MCF-7-ADR cell lines **A.** Western blot analysis of GSK3/β-catenin signaling following treatment with 0.1–10 μM of CRT0066101. Blots are representative of five independent experiments. β-actin was used as the loading control. **B.** Tumorsphere cells were treated with distilled water (control) or 1 μM or 5 μM CRT0066101. Representative confocal images of tumorsphere formation were captured using an Olympus IX71 at a magnification of 400×. Scale bars represent 50 μm. The graph shows the numbers of tumorspheres per 2000 cells. **C.** Analysis of cell-surface expression of CD44+/CD24- on MCF-7-ADR cells following CRT0066101 treatment. Histograms represent results of three independent experiments. Percentages indicate the number of cells in that quadrant. * *p* < 0.001; ** *p* < 0.0001; *** *p* < 0.0001.

The processes associated with PKD/PKCμ phosphorylation were identified as two possible activation pathways: protein kinase C (PKC)-dependent phosphorylation (Ser744/Ser748) and autophosphorylation (Ser916). For full activation, autophosphorylation should occur immediately following PKC-dependent phosphorylation [10, 11]. CRT0066101 is an inhibitor that targets PKD autophosphorylation [16]. To determine the role of PKD/PKCμ autophosphorylation, MCF-7-ADR cells were treated with 1 μM or 5 μM CRT0066101. Western blotting revealed that CRT0066101 inhibited phosphorylation of PKD/PKCμ and GSK3/β-catenin in MCF-7-ADR cells (Figure 3A). However, GSK3/β-catenin signaling was not influenced by CRT0066101 treatment in MCF-7 cells (Supplementary Figure 6A). The number of tumorspheres (>70 μm) following CRT0066101 treatment (1 μM or 5 μM) decreased in a dose-dependent manner relative to the control (Figure 3B). As expected, the CD44+/CD24- population (Q1) also decreased following treatment with 1 μM CRT0066101 (Figure 3C). These results indicated that regulation of breast cancer stemness is necessary for PKD/PKCμ autophosphorylation through GSK3/β-catenin signaling.

### PRKD1 restores drug-resistance

**Figure 4:**
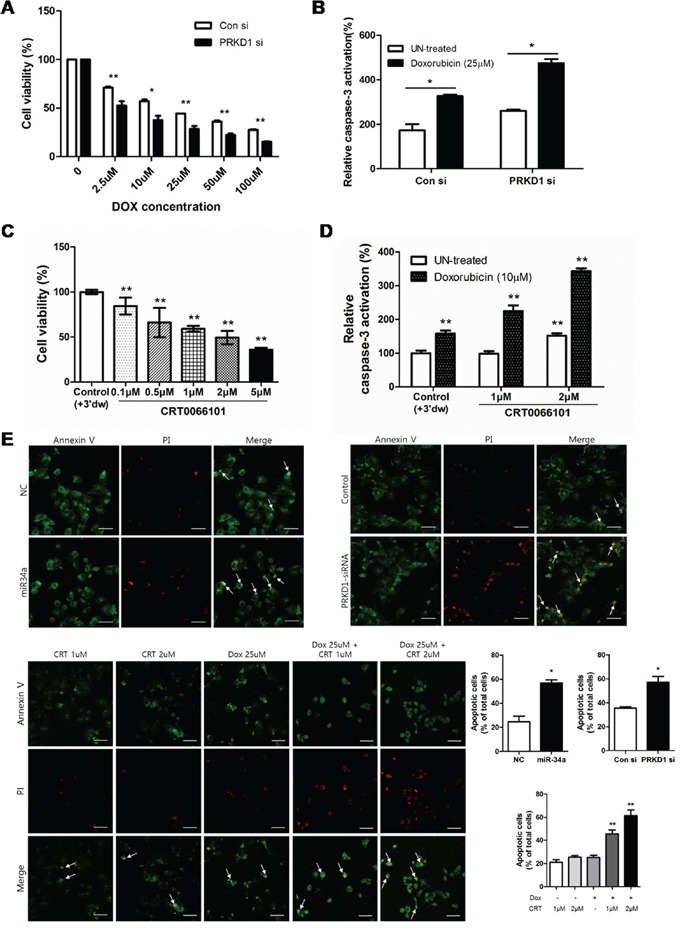
Influence of PKCμ inhibition on MCF-7-ADR cell apoptosis **A.** Cell-viability percentages were detected following transfection of MCF-7-ADR cells with control vector or *PRKD1* siRNA. Cells were treated with doxorubicin after knockdown of *PRKD1* expression in a dose-dependent manner. **B.** The level of caspase-3 activity measured following *PRKD1* downregulation and *PRKD1* downregulation following doxorubicin treatment. **C.** WST-8 assay following CRT0066101 treatment (0.1–5 μM) and 70-h incubation in MCF-7-ADR cells. Optical density was measured at 450 nm. **D.** Caspase-3 activation measured with a colorimetric assay following treatment with 1 μM or 2 μM CRT0066101, or 10 μM doxorubicin. Relative caspase-3 activities were measured at 405 nm. **E.** Representative images of annexin V/PI-stained cells captured by confocal microscopy at a magnification of 200×. Scale bars represent 50 μm. Data represent the mean ± S.D. * *p* < 0.05; ** *p* < 0.001.

Previous reports showed that PKD/PKCμ is involved in apoptosis through caspase-3 inhibition [22]. Therefore, we determined whether *PRKD1* inhibition activates apoptosis in MCF-7-ADR cells, resulting in further reduction in breast cancer stemness. As shown in Figure 4A, exposure of MCF-7-ADR cells to doxorubicin (DOX) resulted in decreased cell survival rates in a dose-dependent manner. Importantly, *PRKD1* knockdown intensified the level of cell death as compared to control. To determine whether decreased cell survival was due to apoptosis, we measured casapase-3 activation. The results indicated that *PRKD1* inhibition resulted in higher caspase-3 activity relative to control. Furthermore, *PRKD1* knockdown following DOX treatment enhanced caspase-3 activity relative to control cells treated with DOX (Figure 4B). Additionally, *PRKD1* inhibition by 0.1–5 μM CRT0066101 decreased cell-viability percentages in a dose-dependent manner in MCF-7-ADR cells (Figure 4C), but cell viability rate was not relevant to treatment of CRT0066101 in MCF-7 cells (Supplementary Figure 6B). CRT0066101-treated cells combined with doxorubicin-induced apoptosis decreased cell viability to a greater degree than CRT0066101 treatment only (Figure 4D). We also performed annexin V and propidium iodide (PI) staining to confirm that *PRKD1* knockdown or inhibition of PKD/PKCμ phosphorylation enhanced apoptosis. The results showed that apoptosis initiation in miR-34a-precursor-treated, *PRKD1* siRNA-treated, and DOX-treated cells increased relative to that in controls. Interestingly, the apoptosis levels increased following CRT0066101 treatment of DOX-treated cells (Figure 4E). Together, these data indicated that downregulated *PRKD1* or inhibition of PKD/PKCμ autophosphorylation restored drug-resistance in MCF-7-ADR cells.

### PKCμ functional inhibition or downregulation suppresses tumor growth in xenograft models

**Figure 5:**
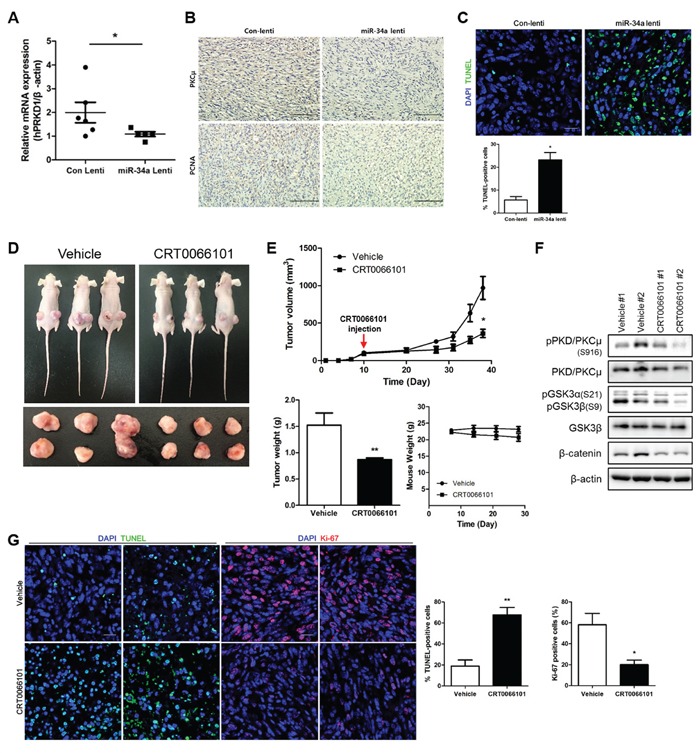
PKCμ downregulation suppresses tumor formation in xenograft models **A.** *PRKD1* mRNA expression was quantified by qRT-PCR from control and miR-34a overexpressed tumors. **B.** Immunohistochemistry was performed to detect the expression of PKCμ and PCNA in control and miR-34a-overexpressed tumors. Magnification at 200×; scale bars represent 10 μm. **C.** TUNEL assay and DAPI staining was performed. Magnification at 400×; scale bars represent 20 μm. **D.** CRT0066101 (65 mg/kg) orally administered to treat established tumors in nude mice daily for 4 weeks. Representative xenografts from three mice. **E.** Decrease in tumor volumes of CRT0066101-treated tumors relative to control tumors. However, any alterations weren't detected in weight of mice. **F.** Western blot analysis of phosphorylated *PRKD1* and GSK3/β-catenin signaling. β-actin was used as the loading control. **G.** Immunofluorescence images showing TUNEL assay and Ki67 in control and CRT0066101-treated tumors. Data are presented as means ± S.D. * *p* < 0.05, ** *p* < 0.001.

In our previous study, we confirmed that miR-34a suppressed *NOTCH1* expression, leading to inhibition of tumor formation in nude mice [5]. Here, we determined whether *PRKD1* downregulation by miR-34a would suppress tumor growth in xenograft models. The level of *PRKD1* expression was downregulated in miR-34a-overexpressed tumors relative to control tumors (Figure 5A). Immunohistochemistry (IHC) staining showed that PKCμ and proliferating cell nuclear antigen (PCNA) were decreased in miR-34a-overexpressed tumors (Figure 5B). To determine whether *PRKD1* suppression by miR-34a would repress tumor growth via apoptosis, we performed a terminal deoxynucleotidyl transferase dUTP nick-end labeling (TUNEL) assay. The results revealed that miR-34a-overexpressed tumors contained higher numbers of apoptotic cells as compared to control tumors (Figure 5C). To further evaluate the effects of PKD/PKCμ functional inhibition, we orally treated mice with established tumors in xenograft models of MCF-7-ADR cells with a dose of 65 mg/kg CRT0066101 daily for 4 weeks. The size of tumors in mice treated with CRT0066101 decreased relative to untreated controls (Figure 5D). As expected, the weight of tumors from mice treated with CRT0066101 decreased as compared to that of control tumors. CRT0066101 treatment didn't cause side effects such as remarkable signs of toxicity and loss of weight in all the animals. (Figure 5E). Moreover, downregulation of phosphorylated PKD/PKCμ through GSK3/β-catenin signaling was also confirmed by western blot analysis (Figure 5F). Finally, we performed TUNEL assay and Ki-67 staining to confirm CRT0066101 enhanced apoptosis and repressed proliferation. The results indicated that CRT0066101 treated tumors were increased apoptosis and decreased proliferation relative to controls (Figure 5G). Altogether, PKCμ inhibition by miR-34a or CRT0066101 contributed to reduction of tumor growth through apoptosis initiation *in vivo*.

## DISCUSSION

*PRKD1* is involved in cell proliferation, apoptosis, cell adhesion, invasion, and vesicle trafficking [23]. Interestingly, *PRKD1* expression exhibits different pattern in various cancer cell types and displays dual functions as an oncogene or tumor suppressor [24]. *PRKD1* expression is downregulated in invasive human breast tumors as compared with normal breast tissue [25]. Similar expression patterns were confirmed by microarray analysis and invasive cellular models, such as SK-BR-3, T-47D, and MDA-MB-231 [25, 26]. Furthermore, reversion of *PRKD1* promoter methylation blocks breast cancer cell invasion and metastasis [27]. Our results showed that patterns of *PRKD1* expression increased drug resistance in MCF-7-ADR cell lines. *PRKD1* was highly expressed in drug resistance cell lines including doxorubicin-resistance MCF-7-ADR cells, tamoxifen-resistance LCC2 cells, and tamoxifen and fluvestrance resistance LCC9 cells (data were not shown). Therefore, we conclude *PRKD1* expression is associated with drug resistance properties. We investigated the expression of miR-34a and *PRKD1* in TCGA data sets (Supplementary Figure 7A). Since *PRKD1* is highly expressed in drug-resistance breast cancer cells, we couldn't find the inverse correlation between miR-34a and *PRKD1* expression in TCGA DB. We further confirmed overall survival according to level of *PRKD1* expression in TCGA clinical data sets (Supplementary Figure 7B). This graph represents patients with high *PRKD1* expression had poor survival than those with low expression. Although we couldn't confirmed that inverse correlation between miR-34a and *PRKD1* expression in whole breast cancer samples, we derived *PRKD1* expression correlates with worse prognosis of breast cancer patients. Furthermore, we confirmed that downregulated *PRKD1* altered apoptosis signaling. Therefore, we suggest that *PRKD1* may be a potential option for the restoration of drug sensitivity in breast cancer cells.

The microRNA miR-34a plays a key role in suppressing tumor. Previous studies reported that miR-34a inhibits CSC function in various cancer types, including prostate cancer [28], pancreatic cancer [29], medulloblastomas [30], and glioblastomas [31]. This molecule also suppresses targets associated with the cell cycle, differentiation, and apoptosis, while inhibiting cancer-cell viability, cancer stemness, metastasis, and chemoresistance [17]. Here, we confirmed that miR-34a negatively regulates *PRKD1* in MCF-7-ADR cells. Additionally, we found that *PRKD1* is a novel target of miR-34a through its binding to the *PRKD1* 3′-UTR. Furthermore, we established that miR-34a-*PRKD1* interactions play a critical role in overcoming cancer stemness and drug resistance in breast cancer cell lines. Prior studies reported that *PRKD1* phosphorylates β-catenin at Thr112/Thr120 and that *PRKD1* overexpression resulted in suppression of β-catenin-mediated transcriptional activity [32]. Phosphorylation of β-catenin is occurs through GSK3, which targets β-catenin as part of the Wnt-signaling protein complex [33]. Furthermore, GSK3β is a kinase involved in prostate cancer stemness and migration through a Wnt-independent mechanism [34]. In our study, we observed that downregulated *PRKD1* led to suppression of the self-renewal capacity of BCSCs through alteration of GSK3/β-catenin signaling. Therefore, these results indicate that *PRKD1* activates breast cancer stemness through GSK3/β-catenin signaling.

Harikumar et al. discovered CRT0066101 as a specific inhibitor of all PKD isoforms [16], showing that CRT0066101 blocks pancreatic cancer growth by inhibiting *PRKD1* autophosphorylation [16]. Here, we confirmed that *PRKD1* activation was blocked by CRT0066101 treatment of breast cancer cell lines and xenograft models. These results provide the first evidence that CRT0066101 may constitute a potential therapeutic agent for breast cancer patients.

**Figure 6:**
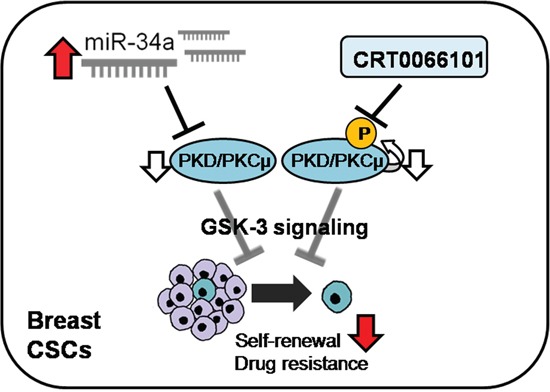
Hypothetical schematic pathway miR-34a directly suppresses *PRKD1* and CRT0066101 inhibits autophosphorylated *PKD/PKCμ*. The two directions represent differential regulation of self-renewal capacity in BCSC and drug resistance in MCF-7-ADR cells through GSK3/β-catenin signaling.

In this study, we showed that *PRKD1* overexpression in MCF-7-ADR cell lines was negatively correlated with miR-34a overexpression. We confirmed miR-34a binding to the *PRKD1* 3′-UTR, resulting in suppression of cancer stemness in BCSCs through the GSK3/β-catenin signaling pathway. Furthermore, we reported that CRT0066101, a known *PRKD1* inhibitor, influenced reductions in BCSC population and drug resistance through the GSK3/β-catenin signaling pathway (Figure 6). Furthermore, we observed that ectopic expression of miR-34a and CRT0066101 treatment prevented tumor growth in xenograft models. In conclusion, *PRKD1* is negatively regulated by miR-34a, leading to suppression of cancer stemness and drug resistance in breast cancer cell lines. These findings provide evidence that *PRKD1* is a key molecule that activates breast cancer stemness and drug resistance, and promotes it as a potential therapeutic target in breast cancer.

## MATERIALS AND METHODS

### Chemicals and reagents

CRT0066101 was purchased from R&D Systems (Minneapolis, MN, USA); the drug was resuspended in autoclaved distilled water for *in vivo* studies. For CRT0066101 treatment, MCF-7-ADR cells (American Type Culture Collection, Manassas, VA, USA) were seeded and 0.1–10 μM CRT0066101 was added, followed by incubation for 1 h. WST-8 was purchased from Enzo Life Sciences, Inc. (Farmingdale, NY, USA). *PRKD1* siRNA and scrambled siRNA (Santa Cruz Biotechnology, Santa Cruz, CA, USA) were transfected using Lipofectamine RNAiMAX (Invitrogen, Carlsbad, CA, USA).

### Cell culture and transfection

The human breast adenocarcinoma MCF7, MCF-7-ADR, and MDA-MB-231 cell lines (American Type Culture Collection) were grown in Dulbecco's modified Eagle's Medium (DMEM; Welgene, Daejeon, South Korea) and supplemented with 10% fetal bovine serum (FBS; Welgene) and 1% penicillin streptomycin in a 37°C humidified incubator under 5% CO_2_. MCF-7-ADR cells were seeded on 10-cm plates in media lacking antibiotics in preparation for RNAi transfection. After 24 h, the cells were transfected with *PRKD1* siRNA using Lipofectamine RNAiMAX (Invitrogen). After 48 h, the cells were collected for western blot analysis or were resuspended in mammosphere medium. For microRNA transfection, MCF-7-ADR cells were seeded for 48 h with miRNA precursors (miR-34a/b/c) using siPORT NeoFX Transfection Agent (Ambion; Thermo Fisher, St. Louis, MO, USA). The miRNA precursors and negative-control precursors were also obtained from Thermo Fisher.

### Quantitative reverse-transcription PCR (qRT-PCR)

The qRT-PCR was performed using a RG3000 instrument (Corbett Robotics, San Francisco, CA, USA) with the SYBR Green-based procedure according to manufacturer instructions. The ABI-7500 instrument (Thermo Fisher) was used to assess *PRKD1* expression in various breast-cancer cell lines. All oligonucleotide primers were designed using DNASTAR (Madison, WI, USA). All qRT-PCR graphs were obtained using comparative C_t_ (ΔΔC_t_).

### Western blotting and antibodies

A total of 30 μg of protein extract was separated by 8% SDS-PAGE and the proteins were electrotransferred to PVDF membrane. The primary antibodies used were: phosphorylated PKD/PKCμ (Ser916), GSK3β, phosphorylated GSK3α (Ser21)/β (Ser9), and β-catenin were purchased from Cell Signaling Technology (Danvers, MA, USA) and the antibody against PKD/PKCμ was obtained from Santa Cruz Biotechnology. β-Actin (Bethyl Laboratories, Montgomery, TX, USA) was used as the loading control. The membranes were washed with 1× PBS/0.1% Tween 20, and bound proteins were detected by enhanced chemiluminescence (Amersham Pharmacia Biotech, Parsippany, NJ, USA).

### Luciferase assay

The 3′-UTR reporter constructs for *PRKD1* were cloned into a pGL3-Control vector and the 3′-UTRs of *PRKD1* were amplified from the genomic DNA of HEK293T cells. The seed sequences of miR-34 from *PRKD1* were mutated using PCR-based methods and the reporter constructs were verified by sequencing. HEK293T cells were transiently transfected with 3′-UTR reporter constructs (1.5 μg/well in 6-well plates) and 15 nM of miR-34 family precursors (Ambion), using Lipofectamine 2000 (Invitrogen). The activity of 3′-UTR reporter constructs was normalized to the activity of the cotransfected pCMV-hRL (40 ng/well in 6-well plates, Promega). After a 24-h incubation, cells were lysed using 1× passive lysis buffer and activity was measured using the Dual Luciferase Assay kit (Promega) according to manufacturer instructions.

### Tumorsphere formation assay (TSA)

For the tumorsphere culture, cells (2000 cells/mL) were cultured in suspension in serum-free DMEM/F12 (welGENE) supplemented with 1% penicillin, B27 (1:50; Gibco; Thermo Fisher), 20 ng/mL epidermal growth factor (Prospec, East Brunswick, NJ, USA), 5 mg/mL insulin (Sigma-Aldrich, St. Louis, MO, USA), and 0.4% bovine serum albumin (Sigma-Aldrich). After ∼10 days, plates were analyzed for tumorsphere formation and were quantified using a microscope (Olympus IX71; Olympus, Tokyo, Japan). For counting tumorspheres, MCF-7-ADR cells were filtered through a 70-μm pore strainer (BD Biosciences, East Rutherford, NJ, USA) and then quantified. CRT0066101 treatment was administered at culture days 6 and 8.

### Surface marker analysis by flow cytometry

We assessed expression of CD44+/CD24- surface markers by collecting cells after transfection with *PRKD1* RNAi or treatment with CRT0066101. Cells were washed with PBS with 2% FBS, stained with anti-CD44 (APC-conjugated; BD Biosciences) and anti-CD24 (PE-conjugated; BD Biosciences) in PBS with 2% FBS, and incubated on ice in the dark for 30 min. Cells were washed again with cold PBS buffer and analyzed by flow cytometry after loading >10,000 cells into a BD CantoII flow cytometer (BD Biosciences) using FACSDiVa software (BD Biosciences).

### Cell-viability assay

MCF-7-ADR cells were plated in 24-well plates and incubated with various concentrations (0.1, 0.5, 1, 5, 10 μM) of CRT0066101 for 72 h. Cell viability was determined by a WST-8 assay (Sigma-Aldrich), and optical density measured at 450 nm using a microplate reader.

### Fluorescent immunohistochemistry

Paraffin sectioned slides from control or miR-34a overexpressed tumors and vehicle or CRT0066101 treated tumors were used. Slides were deparaffinized and rehydrated in Histoclear (3-4 times), then passed through a graded ethanol series (100%, 95%, 80%, 70%) in order. Antigen retrieval was performed by dipping the sections into 0.01M citric acid solution (pH6.0) and boiling in the microwave for 15mins. In case of TUNEL assay, In situ cell Death Detection Kit, Fluorescein (Roche, Indianapolis, USA) labeled apoptotic positive cells and Ki-67 primary antibody (Vector Lab, USA) was applied onto the section, and it was incubated in 4°C overnight. Then, the slides were incubated with DAPI and secondary antibody for 2h. Finally, mounted with mounting solution (Dako) and images were captured with a confocal microscope (Zeiss).

### Preparation of breast cancer xenografted mice

All studies involving the use of nude mice were approved by the Animal Care and Use Committee of Yonsei University Medical School (2015-0087) and performed in specific pathogen-free facilities and under conditions in accordance with the Guidelines for the Care and Use of Laboratory Animals of YUMS. Mice were inoculated subcutaneously with 1.5×10^6^ MCF-7-ADR cells into each flank under 150 μL of saline/zoletil/rompun (7:1:1) anesthesia. Mice were randomized into groups (n = 6 per group), and treatment was started 10 days after tumor implantation. CRT0066101 was administered to tumor-bearing animals orally, five times a week for 4 weeks at dose of 1.6mg/kg. From palpable tumor formation until termination, tumor sizes were measured every 3 to 4 days using calipers, and tumor volume was calculated with the following formula: length × width^2^ × 0.5236. Mice were sacrificed in a 7.5% CO_2_ chamber, and tumors were harvested for immunohistochemistry and other analyses.

## SUPPLEMENTARY FIGURES


