# Supplementary material for: A novel miR-34a target, protein kinase D1, stimulates cancer stemness and drug resistance through GSK3/β-catenin signaling in breast cancer
Source: Oncotarget. 2016 Feb 17;7(12):14791–802. doi: 10.18632/oncotarget.7443 (PMC4924752; doi:10.18632/oncotarget.7443)
Supplement: Supplementary file 1 [file oncotarget-07-14791-s001.pdf]

SUPPLEMENTARY FIGURES

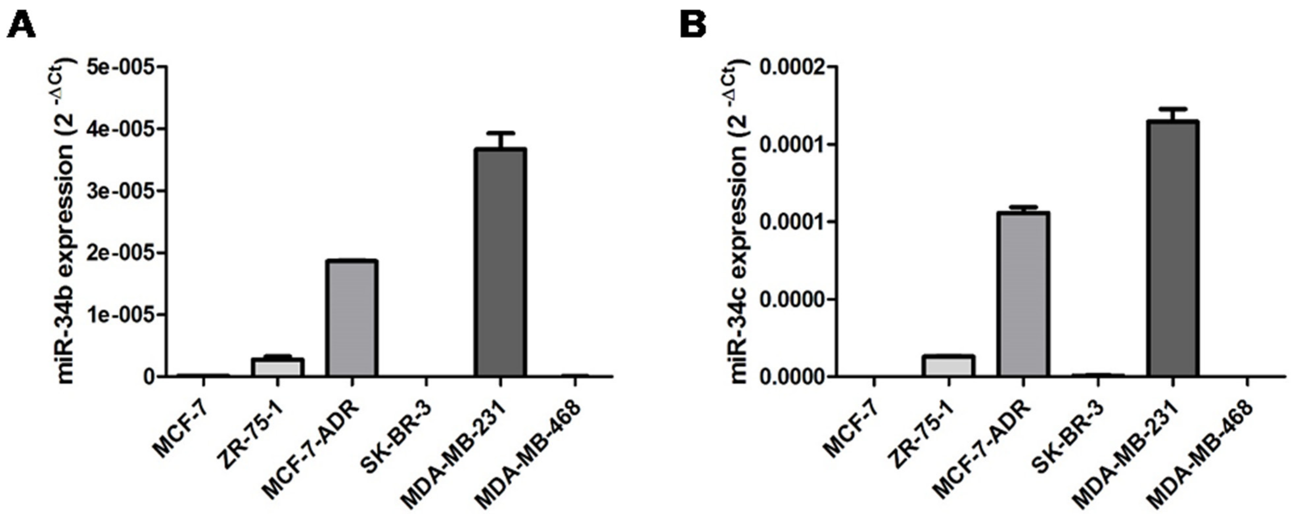

Supplementary Figure S1: No correlation with *PRKD1* expression and miR-34b or miR-34c expression. A-B. Expression levels of miR-34b and miR-34c in various breast cancer cell lines were confirmed by qRT-PCR.

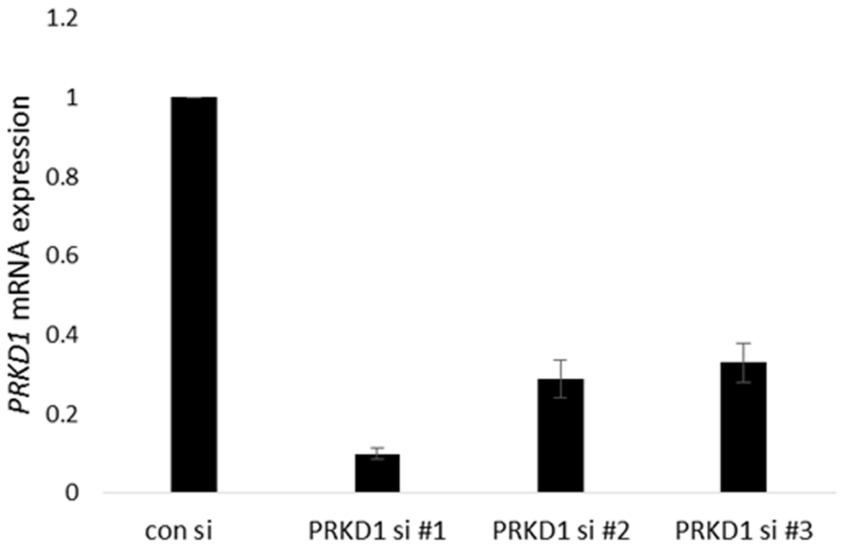

Supplementary Figure S2: Comparison of three different *PRKD1* siRNAs' efficiency.

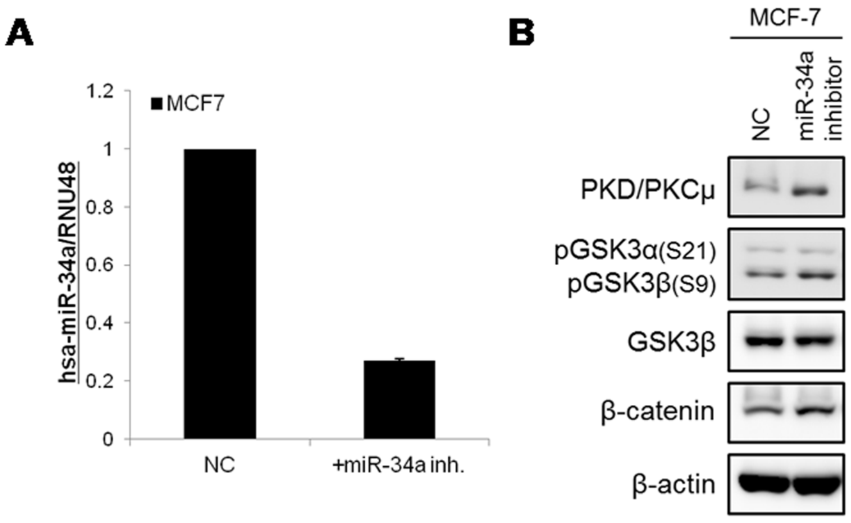

Supplementary Figure S3: Effects of suppressed miR-34a expression in MCF-7 cells. A. Following transfection with miR-34a inhibitors in MCF-7 cells, the expression level of miR-34a were confirmed by qRT-PCR and **B.** GSK3/β-catenin signaling were determined by western blot analysis. β-actin was used as the loading control.

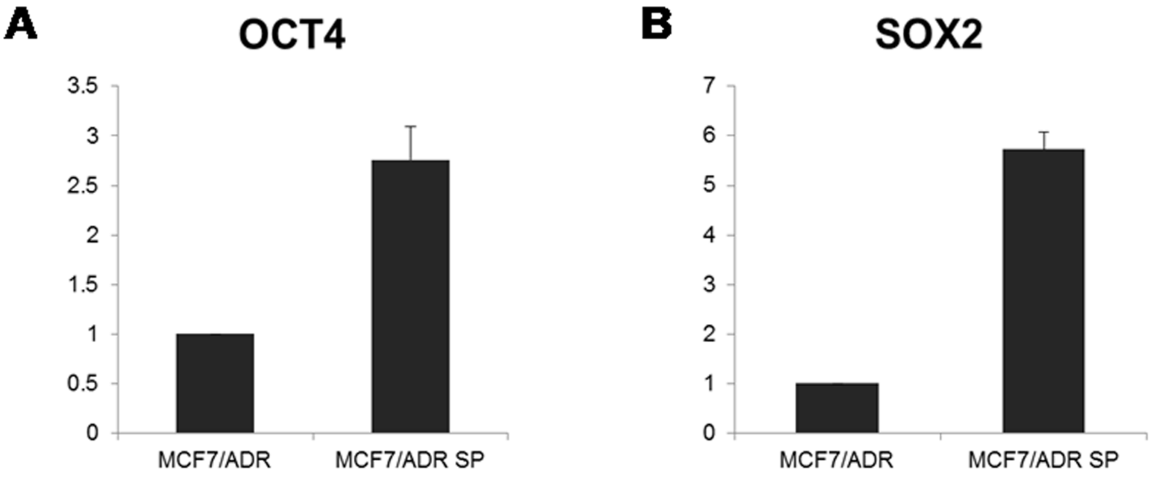

Supplementary Figure S4: The expression level of cancer stemness markers in MCF-7-ADR cells. **A, B.** Expression levels of *OCT4* and *SOX2* were checked by qRT-PCR.

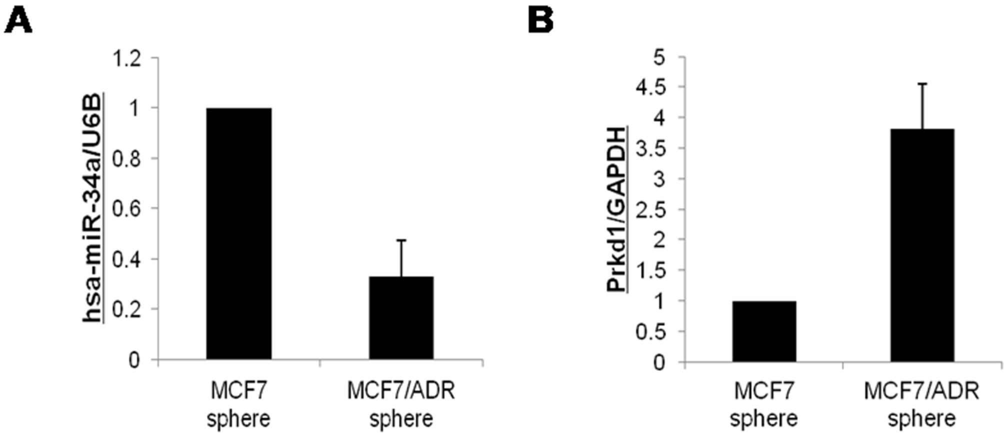

Supplementary Figure S5: The expression level of miR-34a and *PRKD1* in MCF-7 and MCF-7-ADR mammospheres. **A.** miR-34a was highly expressed in MCF-7 cells in sphere status, and **B.** *PRKD1* was highly expressed in MCF-7-ADR mammospheres.

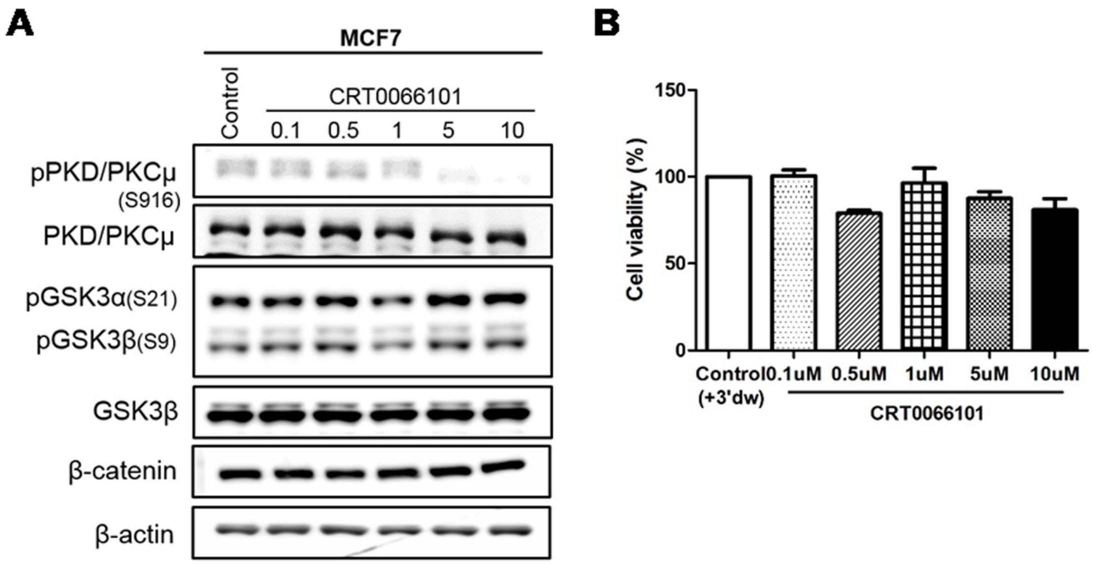

Supplementary Figure S6: No alteration of GSK3/β-catenin signaling and cell viability by treatment of CRT0066101 in MCF-7 cells. A. Western blot analysis revealed the effects of CRT0066101 in MCF-7 cells. **B.** WST-8 assay following CRT0066101 treatment (0.1–5 μM) in MCF-7 cells.

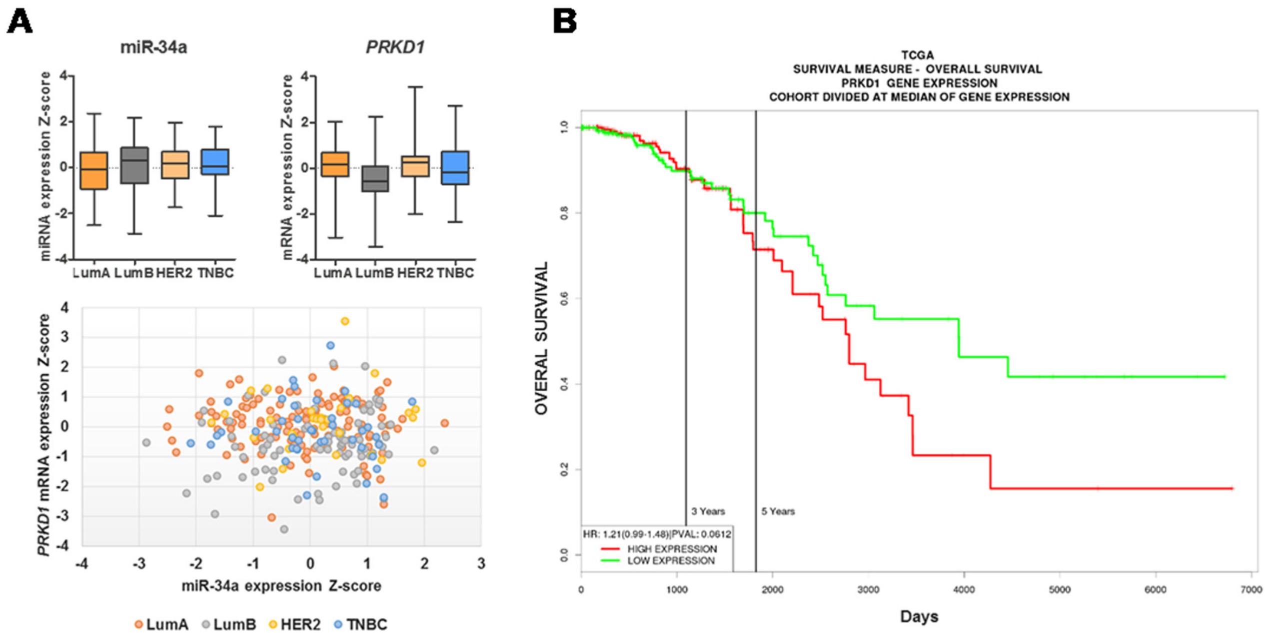

Supplementary Figure S7: Upregulated *PRKD1* expression in correlation with poor prognosis in breast cancer patients. **A.** No correlation between miR-34a and *PRKD1* expression in diverse cell lines **B.** Overall survival according to level of *PRKD1* expression in TCGA clinical data sets.
